# Modulatory Effect of Aerobic Physical Activity on Synaptic Ultrastructure in the Old Mouse Hippocampus

**DOI:** 10.3389/fnagi.2018.00141

**Published:** 2018-05-16

**Authors:** Patrizia Fattoretti, Manuela Malatesta, Barbara Cisterna, Chiara Milanese, Carlo Zancanaro

**Affiliations:** ^1^Cellular Bioenergetics Laboratory, Center for Neurobiology of Aging, Istituto Nazionale di Riposo e Cura per Anziani (INRCA), Ancona, Italy; ^2^Anatomy and Histology Section, Department of Neurosciences, Biomedicine and Movement Sciences, University of Verona, Verona, Italy

**Keywords:** hippocampus, aging, physical activity, exercise, synaptic morphology

## Abstract

Aerobic physical exercise (APE) leads to improved brain functions. To better understand the beneficial effect of APE on the aging brain, a morphometric study was carried out of changes in hippocampal synapses of old (>27 months) Balb/c mice undergoing treadmill training (OTT) for 4 weeks in comparison with old sedentary (OS), middle-aged sedentary (MAS) and middle-aged treadmill training (MATT) mice. The inner molecular layer of the hippocampal dentate gyrus (IMLDG) and the molecular stratum of Ammon’s horn1 neurons (SMCA1) were investigated. The number of synapses per cubic micron of tissue (numeric density, Nv), overall synaptic area per cubic micron of tissue (surface density, Sv), average area of synaptic contact zones (S), and frequency (%) of perforated synapses (PS) were measured in electron micrographs of ethanol-phosphotungstic acid (E-PTA) stained tissue. Data were analyzed with analysis of variance (ANOVA). In IMLDG, an effect of age was found for Nv and Sv, but not S and %PS. Similar results were found for exercise and the interaction of age and exercise. In *post hoc* analysis Nv was higher (60.6% to 75.1%; *p* < 0.001) in MATT vs. MAS, OS and OTT. Sv was higher (32.3% to 54.6%; *p* < 0.001) in MATT vs. MAS, OS and OTT. In SMCA1, age affected Nv, Sv and %PS, but not S. The effect of exercise was significant for Sv only. The interaction of age and exercise was significant for Nv, Sv and %PS. In *post hoc* analysis Nv was lower in OS vs. MAS, MATT and OTT (−26.1% to −32.1%; *p* < 0.038). MAS and OTT were similar. Sv was lower in OS vs. MAS, MATT and OTT (−23.4 to −30.3%, *p* < 0.004). MAS and OTT were similar. PS frequency was higher in OS vs. MAS, MATT and OTT (48.3% to +96.6%, *p* < 0.023). APE positively modulated synaptic structural dynamics in the aging hippocampus, possibly in a region-specific way. The APE-associated reduction in PS frequency in SMCA1 of old mice suggests that an increasing complement of PS is a compensatory phenomenon to maintain synaptic efficacy. In conclusion, the modulation of synaptic plasticity by APE gives quantitative support to the concept that APE protects from neurodegeneration and improves learning and memory in aging.

## Introduction

A large body of work conducted with different laboratory procedures and experimental paradigms have documented that the fully developed central nervous system (CNS) is still capable of significant changes in response to internal and environmental stimuli through plasticity. Physical activity is associated with a positive impact on several physical and mental diseases (e.g., cardiovascular disorders, osteoporosis, depression, anxiety, metabolic syndrome) affecting human beings in adulthood and aging (Ratey and Loehr, 2011; Diaz and Shimbo, 2013; Saunders et al., 2014; Hills et al., 2015). A recent review from Lee et al. (2017) reports that running, a current modality of aerobic physical exercise (APE), provides significant health benefits in preventing chronic disease and premature mortality. With regard to the CNS, several studies reported that APE positively modulates neuronal growth as well as learning and memory (review in Vaynman and Gomez-Pinilla, 2006). In particular, APE is currently considered as a powerful tool to boost brain plastic potential in both adulthood and aging (Smith et al., 2009; Hötting and Röder, 2013; Vilela et al., 2017). Using magnetic resonance imaging, Erickson et al. (2011) have shown that brain function and cognition improve in human beings undergoing aerobic exercise training and found a selective, significant increase of the hippocampal volume in healthy late-adult human volunteers undergoing 1-year aerobic training (selected age range 55–80 years). Human data are supported by magnetic resonance imaging findings in old mice (Mariotti et al., 2014) showing a significant increase in cerebral blood volume of the motor and hippocampal cortex after 30 days of treadmill training.

The hippocampal formation represents a very suitable anatomical model to investigate the neuronal structural dynamics since it is one of the most plastic regions of the CNS. APE has been shown to increase hippocampal neurogenesis in both adult (Eadie et al., 2005; Pereira et al., 2007; Nokia et al., 2016) and aged (van Praag et al., 2005) rats and mice. In addition to benefit hippocampal neurogenesis, physical training induces significant increase in dendritic arborization and synaptic density in old mice and rats (Dietrich et al., 2008; Siette et al., 2013). These findings suggest that neuronal restructuring process underlays the APE-associated changes in the hippocampus found with magnetic resonance imaging in humans (Erickson et al., 2011) and mice (Mariotti et al., 2014). However, detailed morphological investigation of APE-associated changes in synapses is lacking. Because of the lamellar organization of the hippocampal circuitries, the fibers afferent to this region of the CNS do not overlap; accordingly, a morphometric sampling procedure can be referred to discrete areas mostly innervated by specific neurotransmitter systems. For example, the stratum moleculare of the Ammon’s horn 1 pyramidal cells (SMCA1) receives terminal fibers from the perforant path as well as the major terminal field of Ammon’s horn A3 axons, i.e., the Schaffer collaterals. SMCA1 is seriously altered, both structurally and functionally, in physiological and pathological aging (Geinisman et al., 1995; Miller and O’Callaghan, 2003; Small et al., 2004, 2011). Another highly plastic sub-region, the inner molecular layer of the hippocampal dentate gyrus (IMLDG)—a narrow band immediately above the granular cells—is mostly innervated by cholinergic fibers arising in the septum (Kempermann et al., 1997; Schauwecker, 2006). According to the above, SMCA1 and IMLDG appear well suited to study the structural effect of APE interventions in the aging process.

In this study, we hypothesized that APE is able to positively affect the dynamic adaptations of the neuronal terminal zones in aging. The objective of this work was to get quantitative morphological evidence of the effect of APE in old mice. Accordingly, we carried out an ultrastructural quantitative investigation of synapses in the IMLDG and SMCA1 areas of sedentary and physically trained old mice. Comparison was also made of the same areas in middle-aged and middle-aged trained mice.

## Materials and Methods

### Animals

Male middle aged and old Balb/c mice (12 months and 27 months of age, respectively) from the breeding colony of INRCA (Italian National Research Centres on Aging, Ancona, Italy) were used. The animals were housed in groups of four in a temperature (24 ± 1°C) and humidity (50 ± 5%) controlled colony room. All mice were allowed free spontaneous activity in the cage. Light conditions were maintained on 12 h-cycle (lights on at 07:00 AM). Food and water were available *ad libitum*. According to the law in force at the time this study was carried out, the experiment received authorization from the Italian Ministry of Health and was conducted following the principles of the NIH Guide for the Use and Care of Laboratory Animals and the European Community Council (86/609/EEC) directive. A total of 20 mice were used in this study, subdivided in the middle-aged sedentary (MAS, *n* = 5), middle-aged treadmill training (MATT, *n* = 5), old sedentary (OS, *n* = 5), and old treadmill training (OTT, *n* = 5) group. Middle-aged and old mice were assigned to the MAS or MATT and the OS or OTT group at random.

### Treadmill Running

Treadmill running was chosen for APE since exercise parameters such as speed and duration can be strictly controlled thereby allowing a precise exercise load to be set. All of the mice belonging to an experimental group undergo the same protocol and this allows precise monitoring of the performance of each animal. However, treadmill running can induce a certain level of stress in the animal. To minimize stress several provisions were adopted. An experimenter continuously supervised running. Mice were familiarized for 1 week to the treadmill with a single 30-min daily session on the stopped treadmill. During the experiment, mice were transported to the running room 1 h before each daily session of exercise. According to the standard protocol of our laboratory (Mariotti et al., 2014), to prevent injury to the hind limbs provoked by the posterior wall of the treadmill a metal-beaded curtain, as non-noxious stimulus, was used as an incentive; no shock-plate incentive was used.

Treadmill APE was carried out for four consecutive weeks, 5 days a week (Monday to Friday), while resting on weekend. MATT mice had 1-h running/day at belt speed 10 m/min (0% incline). In OTT mice, treadmill APE was adapted to optimize compliance (Fabene et al., 2008); accordingly, each daily running session consisted of 30 min of running at a speed of 8 m/min (0% incline). Mice in the MAS and OS group were daily placed on the stopped treadmill equipment for the same period of time. All mice were weighed at baseline and after the four experimental weeks.

### Morphometry

**Figure 1:**
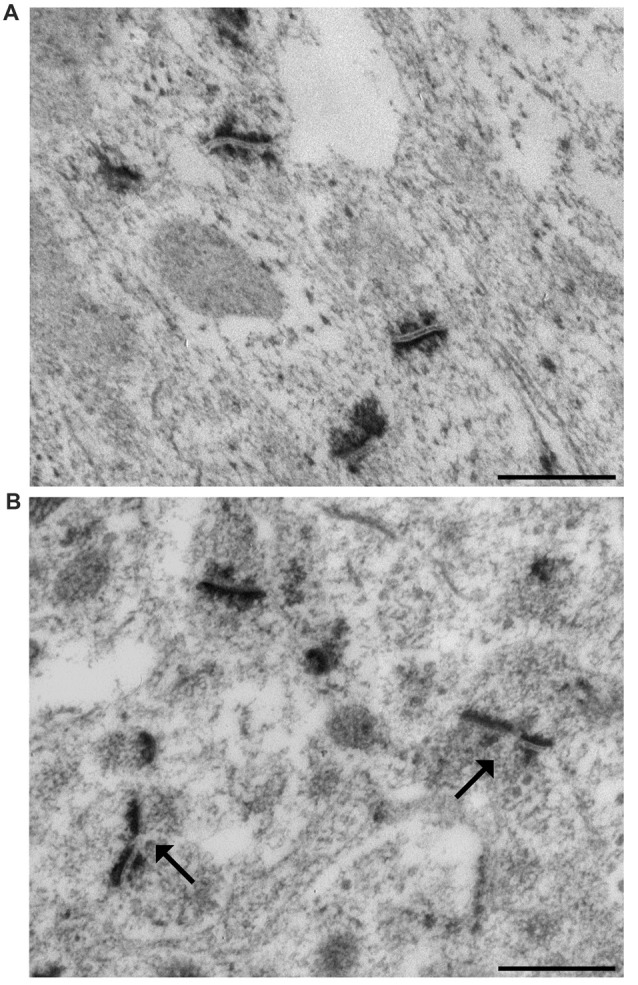
Representative transmission electron micrographs of synapses in the mouse hippocampus. **(A)** The ethanol phosphotungstic acid (E-PTA) technique evidences the synaptic contact zones against an unstained background and enables reliable measurements of synaptic ultrastructural features. **(B)** Arrows indicate two perforated synaptic contacts: pre-synaptic apposition appears as a range of peaks, while the post-synaptic junction appears as a dark uniform line. Bars: 500 nm.

At the end of the 28-day experimental period, mice were anesthetized by intraperitoneal injection of 2,2,2,-tribromoethanol (200 mg/kg body weight) and sacrificed by cervical dislocation. The brain was rapidly removed from the skull. The excised left hippocampi were immediately cut in 0.5 mm thick slices by a tissue chopper. The slices were maintained overnight at 4°C in 2.5% phosphate buffered glutaraldehyde solution and then processed for ethanol-phosphotungstic acid (E-PTA) staining (Bloom and Aghajanian, 1968). Briefly, sections were dehydrated up to 90% ethanol and then incubated in 1% E-PTA solution for 1 h at 60°C. Embedding and sectioning were performed according to standard transmission electron microscopic procedures. Since synaptic staining was very sharp, no contrasting procedure was needed (Figures 1A,B). According to the equal opportunity rule, a systemic random sampling of synaptic contact zones was carried out in the IMLDG and in the SMCA1 with a Kontron KS300 computer-assisted image analysis system equipped with a TV camera directly connected with the electron microscope image plate (Coggeshall and Lekan, 1996; Bertoni-Freddari et al., 2006). One-hundred and fifty fields (9.53 μm^2^ each) per animal were analyzed yielding a total sampled surface of 1429.5 μm^2^/mouse in both IMLDG and SMCA1. The number of synapses/μm^3^ of tissue (numeric density, Nv), the total synaptic area/μm^3^ of tissue (surface density, Sv), and the average size (S) of the contact zones were quantified. Converging data support the idea that an adequate-to-function value of Sv is maintained according to the experiential framework of each individual and that this value is attained through a balance between the selective pruning of redundant contacts and the reinforcement of the most frequently used junctional zones i.e., through an inverse relationship between synaptic number and size. Thus, Nv, S and Sv provide information on discrete aspects of synaptic morphofunctional features and, taken together, they may represent a reliable index of the synaptic plastic adaptation to environmental stimuli (Dyson and Jones, 1984; Hillman and Chen, 1984; Calverley and Jones, 1990). The frequency of a peculiar type of synapses, the perforated synapses (PS) was also calculated as percentage of total synaptic contacts. PS are characterized by discontinuous postsynaptic densities when viewed in transverse section and are larger than non-PS (Figure 1B) and are considered as an index of synapse plasticity and enhanced transmission efficacy in adult CNS (Jones and Harris, 1995; Geinisman, 2000; see below).

### Statistical Analysis

In order to assess the effect of age and exercise on the measured dependent variables, morphometric mean values in the four groups were analyzed using two-way analysis of variance (ANOVA; factor: age, exercise) followed by *post hoc* analysis with Bonferroni correction where needed. One-way ANOVA was used to compare mice weight. Effect size was assessed calculating *η*^2^ or Cohen’s *d* as needed. Following Cohen (1988), we interpret estimated *η*^2^ and *d* values as follows: *η*^2^ = 0.01 small, 0.06 medium, 0.14 large; *d* 0.2 small, 0.5 medium, 0.8 large. Statistical significance was set at *p* < 0.05. The results are expressed as mean ± standard error of the mean (SEM). All analysis was performed using the IBM-SPSS statistical software package v. 23.

## Results

The mean body weight of the four groups of mice at baseline (Time 0) and after the 4-week experimental period (Time 1) is reported in Table 1. No statistically significant difference was found between Time 0 and Time 1 in any group.

**Table 1:** Body weight (g) of the four groups of mice (*n* = 5 each) at baseline (Time 0) and 4 weeks later (Time 1).

Experimental time	MAS	MATT	OS	OTT
Time 0	41.8 ± 0.97	45.0 ± 0.89	39.8 ± 2.35	39.2 ± 0.73
Time 1	42.6 ± 0.68	43.0 ± 1.65	34.0 ± 1.60	37.2 ± 1.77

*Means ± SEM. No significant difference was found at Time 1 vs. Time 0 in any group. MAS, middle-aged sedentary; MATT, middle-aged treadmill training, OS, old sedentary; OTT, old treadmill training*.

The ultrastructural morphology of synapses in IMLDG and SMCA1 from MAS, MATT, OS and OTT mice is shown in Supplementary Figure S1.

Quantitative analysis of electron micrographs yielded the following results.

### IMLDG

**Figure 2:**
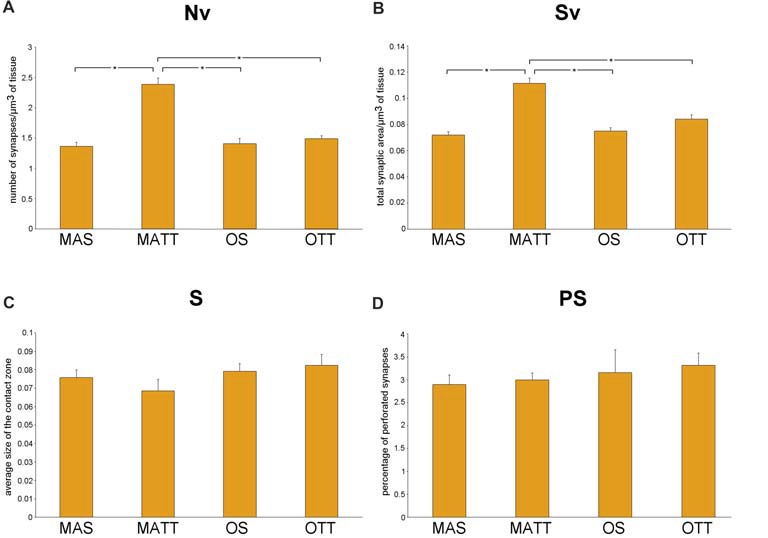
**(A–D)** Mean value ± standard error of the mean (SEM) of synaptic ultrastructural variables measured in the inner molecular layer of dentate gyrus (IMLDG) in middle-aged sedentary (MAS), middle-aged running (MATT), old sedentary (OS) and old treadmill-exposed (OTT) mice. Nv, numeric density of synapses; Sv, surface density of synapses; S, average size of synaptic contacts; PS, percent of perforated synapses. Asterisk indicates between-group statistically significant differences (*p* ≤ 0.05; Bonferroni’s *post hoc* test).

Figure 2 reports the mean ± SEM values of morphometric variables measured in IMLDG of the four groups of mice. Two-way ANOVA (Table 2) showed a significant effect of age for Nv and Sv with a large effect size, but not S and %PS. Similar results were found for exercise and the interaction of age and exercise. *Post hoc* analysis showed significantly higher Nv in MATT vs. MAS, OS and OTT (+75.1%, +69.5, +60.6%; *p* < 0.001 for all; *d* = 4.1, 3.9, 3.6 respectively). *Post hoc* analysis showed that Sv was significantly higher in MATT vs. MAS, OS and OTT (+54.6%, +48.4%, +32.3%, respectively; *p* = < 0.001 for all; *d* = 3.9, 3.6, 2.8 respectively).

**Table 2:** Two-way ANOVA (factor: age, exercise) for several morphometrical synaptic variables in two hippocampal areas of mice. The effect for the interaction of the two factors (age × exercise) is also reported.

Variable	Factor	IMLDG				SMCA1			
		*df*	*F*	*η*^2^	*p*	*df*	*F*	*η*^2^	*p*
Nv	Age	1	29.110	0.645	<0.001	1	10.141	0.338	0.006
	Exercise	1	48.394	0.752	<0.001	1	2.813	0.150	0.113
	Age × exercise	1	35.644	0.690	<0.001	1	7.723	0.326	0.013
Sv	Age	1	14.384	0.473	0.002	1	9.318	0.368	0.008
	Exercise	1	57.345	0.782	<0.001	1	9.109	0.363	0.008
	Age × exercise	1	22.460	0.584	<0.001	1	23.494	0.595	<0.001
S	Age	1	2.657	0.142	0.123	1	1.507	0.086	0.237
	Exercise	1	0.125	0.008	0.728	1	0.711	0.043	0.412
	Age × exercise	1	0.995	0.059	0.333	1	0.003	0.000	0.958
%PS	Age	1	0.848	0.050	0.371	1	15.275	0.488	0.001
	Exercise	1	0.163	0.010	0.692	1	2.159	0.119	0.161
	Age × exercise	1	0.008	0.001	0.929	1	11.115	0.410	0.004

*Nv, numeric density of synapses; Sv, surface density of synapses; S, average size of synaptic contacts; PS, percent of perforated synapses; IMLDG, inner molecular layer of dentate gyrus; SMCA1, stratum moleculare of hippocampal Ammon’s horn1 pyramidal cells*.

### SMCA1

**Figure 3:**
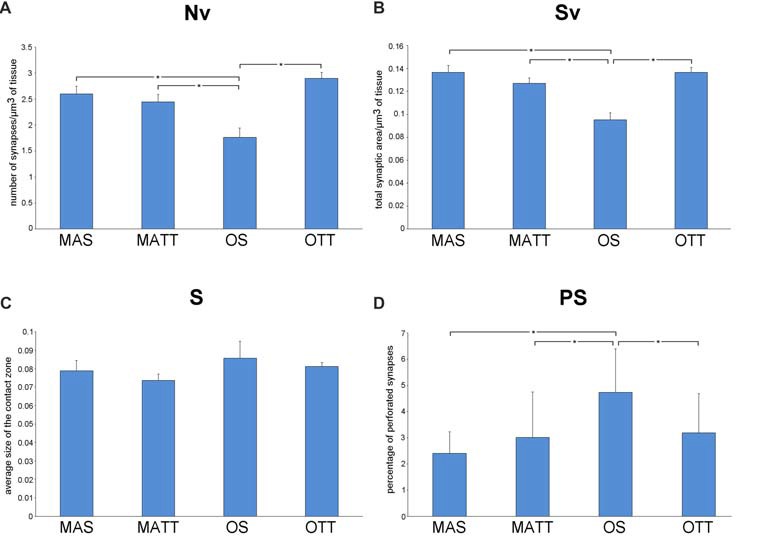
**(A–D)** Mean values ± SEM of synaptic ultrastructural variables measured in stratum moleculare of hippocampal Ammon’s horn1 pyramidal cells (SMCA1) in MAS, middle-aged running (MATT), OS and old treadmill-exposed (OTT) mice. Nv, numeric density of synapses; Sv, surface density of synapses; S, average size of synaptic contacts; PS, percent of perforated synapses. Asterisk indicates between-group statistically significant differences (*p* ≤ 0.05; Bonferroni’s *post hoc* test).

Figure 3 reports the mean ± SEM values of morphometric variables measured in SMCA1 of the four groups of mice. Two-way ANOVA (Table 2) showed that age has a significant effect with a large effect size for Nv, Sv and %PS, but not S. The effect of exercise was significant with a large effect size for Sv and showed a low, non-significant *p* value of 0.113 with a large effect size for Nv. The interaction of age and exercise was significant with a large effect size for Nv, Sv and %PS. *Post hoc* analysis showed that the Nv was significantly lower in OS vs. MAS, MATT and OTT (−32.1%, *p* = 0.004, *d* = 1.9; −27.8, *p* = 0.020, *d* = 1.5; −26.1, *p* = 0.037, *d* = 1.4 respectively). No significant difference was found between MAS and OTT (−8.07%). *Post hoc* analysis showed that Sv was significantly lower in OS vs. MAS, MATT and OTT (−30.3%, *p* = 0.001, *d* = 2.5; −23.4, *p* = 0.003, *d* = 1.9; −29.2 *p* < 0.001, *d* = 2.5 respectively). MAS and OTT were not significantly different (−0.14%). *Post hoc* analysis showed significantly higher PS frequency in OS vs. MAS, MATT and OTT (+96.6%, *p* = 0.001, *d* = 2.3; +54.7%, *p* = 0.009, *d* = 1.7; +48.3%, *p* = 0.022, *d* = 1.5).

## Discussion

In this work, ultrastructural morphometric analysis of synapses was carried out in two regions of the mouse hippocampus to assess the structural effects of APE in old mice. Results demonstrate the following points:

1. Either age and APE affect the density of synapses in both IMLDG and SMCA1 hippocampal areas while their average contact area remains similar; however, APE seems to act differently in the two areas of middle-aged mice;
2. APE shows a positive effect in old mice by increasing synaptic density, this effect being more prominent in SMCA1;
3. In SMCA1, APE is able to revert the age-associated increase in the percentage of PS.

We report herein that APE is able to significantly increase Nv and Sv in IMLDG in middle-aged but not old mice (Figure 2). Our findings on the effect of exercise in the middle-aged mouse IMLDG are in agreement with several works reporting increased synaptic density in the dentate gyrus after exercise associated with increased neurogenesis and neurotrophic factors (Eadie et al., 2005; Pereira et al., 2007; Nokia et al., 2016). In the old mouse, it can be hypothesized that the process going from stimulation of progenitor cells to formation of mature and functioning neurons is delayed and the newborn granule cells were not able, in the 4-week experimental period, to form new synaptic contacts (van Praag et al., 2002; Rao et al., 2005). In SMCA1, aging was associated with a reduction of Nv and Sv and an increase of %PS, while S did not change. APE affected Sv only. The interaction of age and exercise was significant for Nv, Sv and %PS. It should be underlined that the morphology of SMCA1 is more susceptible to age-induced modification than that of IMLDG as far as synapses are concerned. This is not surprising, because CA1 is extremely vulnerable to age (Miller and O’Callaghan, 2003) and it is the most severely damaged hippocampal zone in Alzheimer’s disease (West et al., 2004), and in ischemic events (Kang et al., 2002; Small et al., 2004). Moderate APE revealed able to revert Nv, Sv and %PS values of old mice to those of the middle-aged. Instead, in middle-aged mice physical exercise had no effect because there was no neurogenesis and the neuronal population (and consequently the synaptic contacts) remained constant during the 4 weeks of physical activity. However, in rat SMCA1 synaptic density decreases with age (Bertoni-Freddari et al., 1999; Balietti et al., 2008). The APE-associated formation of new synaptic contacts in old age is probably due to improved cardiovascular function, energy metabolism and reduction of inflammation. Finally, the mean value of S was similar for MAS, OS, MATT and OTT mice in both IMLDG and SMCA1 (Figures 2C, 3C) with group mean values ranging close to one another in each area. This finding suggests that aging had not a major effect on synapse size and APE did not affect this structural variable. Taken together, the above findings strongly suggest that APE is able to increase the total synaptic area per volume of tissue in old mice via an increased number of junctions while leaving unaffected junction size. The frequency of PS was significantly different in the four groups in SMCA1 (Figure 3D) but not IMLDG (Figure 2D). In the former, PS were not affected by APE in the middle-aged mouse, significantly increased with aging and decreased with APE to a mean value similar to the middle-aged. Accordingly, data presented herein show a positive effect of APE in the hippocampus even in old age and suggest that hippocampal regions are not uniformly influenced by physical training. This suggestion is supported by consistent data from other authors (Hillman et al., 2008; Ahlskog et al., 2011; Erickson et al., 2011, 2012; Intlekofer and Cotman, 2013).

We found that synaptic plasticity in SMCA1 is more influenced by exercise than in IMLDG (Figures 2D, 3D). The reasons for such a difference are probably to be found in region-peculiar features of these two zones. An on-going neurogenesis has been reported to occur in the dentate gyrus of adult individuals (Zhao et al., 2008); this implies that the population of granule cells is composed of differentiated and newborn elements in this hippocampal region. Exercise boosts this proliferation of new granule cells (van Praag et al., 1999a,b; Kronenberg et al., 2006) by increasing the proportion of elements bearing immature neuronal properties (Piatti et al., 2006). The functional maturation of these newborn cells is attained in 6–8 weeks (Mongiat and Schinder, 2011). Therefore, the lack of measurable ultrastructural changes at the synaptic terminals of the dentate gyrus granule cells in OTT mice may be due to the presence of a consistent number of newborn granule cells not yet contributing to synaptic rearrangements (Patten et al., 2015; Vivar and van Praag, 2017). In contrast to the dentate gyrus, in hippocampal CA1 no neurogenesis has been reported to occur as a consequence of exercise. Accordingly, the quantitative synaptic changes shown in SMCA1 could take place in a fixed number of fully differentiated neurons (Patten et al., 2015). Our findings are to some extent at variance with those reported by Siette et al. (2013) in rats. Investigating female Fischer 344 rats subjected to voluntary wheel exercise for 12 weeks, Siette et al. (2013) reported that aged non-runner rats have lower synaptophisin density in comparison to young non-runners across most hippocampal areas i.e., the dentate gyrus polymorph layer, the inner molecular layer, the CA3 and CA2 area, and the CA1 radiatum. Voluntary running counteracted age-related pre-synaptic loss by increasing synaptophisin density across the hippocampal subregions in aged rats. Moreover, voluntary wheel running “did not alter presynaptic density estimates in any hippocampal subregion in younger rats”. Our results were in agreement with those of Siette et al. (2013) as far as the SMCA1 region is concerned; instead, we found significant higher synaptic density in the IMLDG of MATT mice. This discrepancy may be due to differences in the species used (rat vs. mouse), gender (female vs. male), and breed as well as the different experimental paradigm (voluntary wheel running vs. treadmill running) and the age of animals (24-month-old and >28 months at the time of sacrifice). Moreover, the synaptic assay was different (synaptophisin immunohistochemistry vs. ultrastructural morphometry) and the duration of training was much longer in the Siette et al. (2013) work i.e., 12 weeks vs. 4 weeks).

The findings on PS frequency reported herein deserve a specific comment. Despite the presence of aligned discontinuities (gaps) in their postsynaptic and presynaptic densities, PS are considered a single contact due to the presence of a single presynaptic terminal with a unitary population of synaptic vesicles. Although the biological/functional significance of PS is still debated, current reports on this topic converge on two major tenets: PS represent an intermediate step in synaptic remodeling (Geinisman et al., 1995; Geinisman, 2000) or they are a “separate population” of junctional areas (Jones and Calverley, 1992; Jones and Harris, 1995). In the former hypothesis, synaptic morphology undergoes, following stimulation, an ordered sequence of steps that include: (i) enlargement of a non-perforated junction; (ii) fragmentation into smaller clods; and (iii) splitting into daughter synapses. In the latter hypothesis, PS are a more permanent type of synaptic connection indicative of synaptic remodeling and turnover in the mature CNS. No clear-cut evidence has been presented so far to confirm or disprove these two assumptions that, however, are not mutually exclusive. The supporters of both hypotheses agree that: (i) PS may represent the most significant key-hallmark of synaptic plasticity in the fully differentiated adult CNS; (ii) increasing PS frequency enhances the efficacy of synaptic transmission; and (iii) PS are an adaptive response able to expand the size of the contact zones, thereby increasing synaptic efficacy (Jones and Harris, 1995; Geinisman, 2000). According to the above considerations the current findings on PS frequency (Figure 3D) argue for a modulatory effect of APE on synaptic plasticity in the SMCA1 of old mice and lend support to the “separate population hypothesis” of PS. As shown in Figure 3, Nv and PS frequency show opposite patterns in OTT vs. OS mice (Nv, +35.0%; PS, −32.6%). This is apparently not due to the splitting of larger contacts, since S is the same in both OTT and OS. On the contrary, in middle-aged mice physical exercise does not influence the percentage of PS, Nv and Sv. As previously stated, in the adult animal the population of CA1 neurons is stable, not affected by physical activity and this further underlines the difference between the two areas analyzed. According to the “separate population hypothesis”, the lower frequency of PS in OTT together with increased Sv could be due to the formation of new contacts rather than augmentation of the number of PS. Independently of the biological/functional significance of PS, the joint evidence presented herein of changes in PS frequency and Nv provides novel information on the synaptic structural rearrangements as consequence of an environmental stimulating factor. From a morphofunctional standpoint, the question arises of why should an increase in Nv be better suited than an increase in PS frequency to improve the performance of any discrete CNS area undergoing stimulation. Actually, Nv is commonly considered a reliable structural correlate of improved functioning: the higher the Nv the finer the tuning of the communication among neurons; the higher the Nv the wider the properly connected CNS area. Instead, an increase in PS frequency would merely be a compensating phenomenon following the impairment of synaptic function (e.g., the age-related decrease of Nv), leading to augmentation of the overall post-synaptic density (Calverley and Jones, 1990). In this context, the current data support the concept that APE is effective in inducing positive structural and functional changes at the synaptic junctional areas. The reasons for that are probably multifactorial given the complex bodily effects of APE including the general promotion of physiological functions mediated by the improvement in blood flow as well as the facilitation of synaptic plasticity through increased expression of neurotrophins (e.g., brain-derived-neurotrophic-factor; Berchtold et al., 2005). In fact, physically trained laboratory animals show hippocampal neurogenesis, a significant increase in dendritic length, dendritic complexity, spine density and neural progenitor proliferation (Eadie et al., 2005).

Aging is characterized by an impairment of memory function correlated with decreased synaptic density in the hippocampus (Bertoni-Freddari et al., 1996a,b; Platano et al., 2008). Deterioration of hippocampus precedes and leads to memory impairment in late adulthood (Erickson et al., 2011). In particular, the CA1 area is important to encode memories and the dentate gyrus is probably involved in spatial pattern separation. Physical activity has emerged as a promising, low-cost strategy to improving neurocognitive function (Kramer and Erickson, 2007; Gomez-Pinilla and Hillman, 2013), fighting hippocampal impairment, and protecting against memory decline (de Bruijn et al., 2013). As reported above, voluntary wheel exercise increases neurogenesis and cell proliferation in the hippocampus of old rats (van Praag et al., 2005) and enhances synaptic density in young (Dietrich et al., 2008) and old (Xu et al., 2017) mice. Our findings suggest that moderate physical activity in late aging is able to revert SMCA1 synaptic density to the values of the middle-aged group and hippocampal plasticity is retained in aging. Because hippocampal CA1 zone is involved in memory processes, it can be hypothesized that the increased synaptic density is able to improve memory function.

The plasticity we found in hippocampal synapses was apparently independent of body weight, since no statistically significant change was found in body weight of any group of mice after the 4-week experimental period. In particular, body weight did not significantly change after APE, despite a slight decrease in both the MATT and OTT groups. This is probably in relation to the moderate intensity of physical activity used in this work (Chen et al., 1998; Aguiar et al., 2011; Bayod et al., 2011; Lalanza et al., 2012).

In conclusion, this work showed that the synaptic junctional area is an important target for APE effect in old mice. The results presented herein give quantitative support to the growing body of evidence documenting that APE results in protection from neurodegeneration as well as enhancement of learning and memory in aging (Vaynman and Gomez-Pinilla, 2006; Hillman et al., 2008; Intlekofer and Cotman, 2013).

## Author Contributions

PF performed electron microscopy and morphometry, interpreted results, wrote the article. MM performed electron microscopy, interpreted results and wrote the article. BC performed animal experiments, collected data, wrote the article. CM performed animal experiments and wrote the article. CZ conceived the work, interpreted results and wrote the article.

## Conflict of Interest Statement

The authors declare that the research was conducted in the absence of any commercial or financial relationships that could be construed as a potential conflict of interest.

## Abbreviations

APE: aerobic physical exercise
CNS: central nervous system
IMLDG: inner molecular layer of the dentate gyrus
Nv: numeric density of synapses
PS: perforated synapses
S: average size of synaptic contacts
SMCA1: stratum moleculare of the Ammon’s horn 1 pyramidal cells
Sv: surface density of synapses.
