# Supplementary material for: Modulatory Effect of Aerobic Physical Activity on Synaptic Ultrastructure in the Old Mouse Hippocampus
Source: Front Aging Neurosci. 2018 May 16;10:141. doi: 10.3389/fnagi.2018.00141 (PMC5964889; doi:10.3389/fnagi.2018.00141)
Supplement: Supplementary file 1 [file Image_1.PDF]

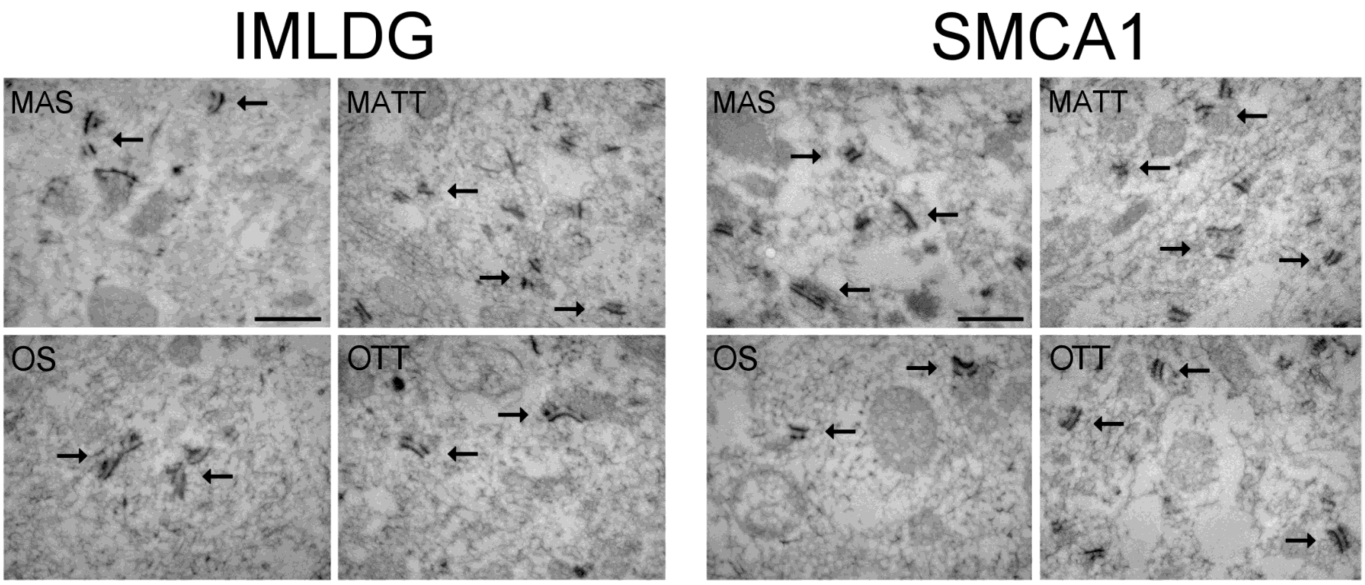

Figure S1. Transmission electron micrographs of IMLDG and SMCA1 areas from middle-aged sedentary (MAS), middle-aged treadmill training (MATT), old sedentary (OS), and old treadmill training (OTT) mice. Arrows indicate synapses stained with the ethanol phosphotungstic acid (E-PTA) technique. Bars: 500 nm.
